# From *kirigami* to three-dimensional paper-based micro-analytical device: cut-and-paste fabrication and mobile app quantitation†

**DOI:** 10.1039/c9ra04014e

**Published:** 2019-07-26

**Authors:** Jianhua Wang, Lishen Zhang, Xiaochun Li, Xiaoliang Zhang, Hua-Zhong Yu

**Affiliations:** College of Biomedical Engineering, Taiyuan University of Technology Taiyuan Shanxi 030024 China lixiaochun@tyut.edu.cn; Department of Chemistry, Simon Fraser University Burnaby British Columbia V5A 1S6 Canada hogan_yu@sfu.ca

## Abstract

Nowadays quantitative chemical analysis is usually costly, instrument-dependent, and time-consuming, which limits its implementation for remote locations and resource-limited regions. Inspired by the ancient papercutting art (*kirigami*), we herein introduce a novel cut-and-paste protocol to fabricate 3D microfluidic paper-based analytical devices (μPADs) that are suitable for on-site quantitative assay applications. The preparation of the device is fast, simple, and independent of any lithographic devices or masks. Particularly designed reaction “channels” were pre-cut from a piece of filter paper, then assembled back to the silanized, superhydrophobic paper pads. The different layers of the device were assembled using a chemically-inert adhesive spray. The fabricated device has high efficiency of liquid handling (up to 60 times faster than conventional methods) and it is particularly inexpensive. Beyond the benchtop fabrication advantage, in conjunction with a custom mobile app developed for colorimetric analysis, we were able to quantify representative environmental contaminants (*i.e.*, the amount of Cr(vi) and nitrite ions) in various water samples with the cut-and-paste μPADs (namely kPADs). Their detection limits (0.7 μg mL^−1^ for Cr(vi) and 0.4 μg mL^−1^ for nitrite ions, respectively) are comparable with conventional spectrophotometric methods, which confirm the potential of kPADs for on-site environmental/sanitary monitoring and food toxin pre-screening.

## Abstract 2

The ancient papercutting art (*kirigami*) inspired a novel cut-and-paste protocol to fabricate paper-based micro-analytical devices for on-site quantitative assays.

## Graphical abstract

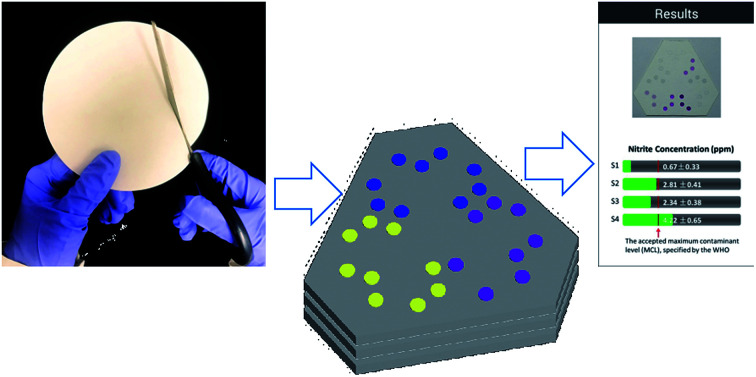

## Introduction

On-site chemical analysis and point-of-care biomedical diagnosis are the frontiers of today's analytical sciences, for which the challenges are the cost of device fabrication and limited success in the performance for quantitation.^1,2^ As one of the best supplements came into attention a decade ago,^3^ microfluidic paper-based analytical devices (μPADs) have attracted extensive attention as reviewed recently in literature.^4–8^ Even compared with microfluidic devices fabricated on traditional substrates (glass, quartz, silicon, metal, and polymer),^9–13^ μPADs are advantageous in terms of cost-effectiveness, excellent biocompatibility, and ease of fabrication/operation. As the newest addition to the family, three-dimensional (3D) μPADs have shown their enhanced analytical performance in pump-free distribution of fluids in both vertical and lateral directions and improved throughput with high-density assays.^4–6,14,15^ With superior performance projected, the fabrication of 3D μPADs, nonetheless, involves a number of cumbersome steps of substrate patterning and device assembling. The patterning step creates hydrophilic reaction “channels” and hydrophobic “barriers” on paper substrates, which is also an essential step in the fabrication of conventional μPADs. Existing patterning methods can be classified in two categories: one is to deposit or react hydrophobic materials (wax,^16,17^ photoresist,^3^ polymer,^17,18^ or organosilane^19^) into designed patterns using specific deposition or printing instruments;^3,16–19^ the other involves the initial functionalization of the entire surface followed by lithographic procedures.^20^ There are limitations in both approaches, *i.e.*, relying on either advanced deposition/printing instrumentation or effective methods of degrading (patterning) the hydrophobic coating (typically prolonged UV irradiation). Particularly for the latter, photochemically activated polymer surfaces tend to regain their hydrophobicity rapidly,^21^ which eventually inhibits the lateral liquid flow across these regions.

For the assembly of 3D μPADs, the challenge is to ensure uninhibited transfer of solutions from one layer to another.^14–16,22,23^ Martinez *et al.* assembled patterned paper substrates with punched double-side tapes (punched and refilled with cellulose powders), to create a prototype 3D device that tests 4 different samples for up to 4 different analytes.^14,21^ Liu and Crooks prepared a device by folding a single piece of paper patterned lithographically for colorimetric and fluorescence assays.^15^ Such a paper-folding (*origami*) approach to fabricate 3D μPADs has been lately expanded to other novel designs, stacking methods, and chemiluminescence immunoassays for real-world samples.^23^ Most recently, Maria *et al.* reported a unique photolithographic method to create perpendicular channels by patterning both sides of the same sheet of photoresist-treated filter paper.^24^ The other method of rapid prototyping of multiplex microfluidic devices (not particularly applicable to μPAD), namely xurography, uses a digital cutting plotter for making microstructures in various polymeric films (with limited exploration of paper substrates).^25^ which has recently shown great advantages in creating PVC micromixers and micro-mixing arrays.^26,27^

Inspired by the ancient papercutting art (*kirigami*),^28^ we introduce herein a cut-and-paste protocol to fabricate 3D μPADs (namely kPADs) that are suitable for on-site quantitative assay applications. The novelty of our approach is beyond the cutting aspect that has been reported previously,^29,30^ rather, on the modification of the pre-cut paper substrate to be superhydrophobic and the reassembly with the untreated, hydrophilic reaction “discs” or “channels” with a chemically inert adhesive spray to prepare the kPADs. While the pioneering *origami* approach to prepare 3D μPADs indeed provided a unique and simplified device assembly protocol,^15,23^ the *kirigami* (particularly cut-and-paste) protocol reported herein aims to solve both assembly and patterning challenges (*vide supra*). In the following sections, we will describe the benchtop fabrication of kPADs with moderate complexity that is entirely instrumentation-free, *i.e.*, they can be created with a pair of scissors and a pack of laboratory filter paper in a standard wet laboratory setting. More importantly, in conjunction with smartphone-reading colorimetric protocol such devices are demonstrated for quantitative detection of environmental contaminants (*i.e.*, Cr(vi) and nitrite) in water samples.

## Materials and methods

### Materials and reagents

Methyltrioctylammonium chloride, 1,5-diphenylcarbazide (1,5-DPC), octadecyltrichlorosilane (OTS), methyltrichlorosilane (MTS), citric acid, and acetone were ordered from Sigma-Aldrich (St. Louis, MO). Hydrochloric acid (HCl), sodium hydroxide (NaOH), *n*-hexane, sulfanilamide, and *N*-(1-naphthyl)ethylenediamine were ordered from Aladdin Reagent Co. Ltd. (Shanghai, China). Potassium chromate (K_2_CrO_4_), sodium nitrite (NaNO_2_), and methanol were purchased from Tianjin Kermel Chemical Reagent Co. Ltd. (Tianjin, China). Filter paper packs (Whatman™, Grade 3 and Grade 4) were purchased from local distributors of GE Healthcare Life Sciences (Beijing, China).

Binary silane solutions were prepared by mixing two organosilanes (OTS/MTS = 7 : 3) in *n*-hexane with a total concentration of 0.2% (v/v) to prepare the hydrophobic hollow pads in the preparation of the kPADs. Stock solutions (1000 μg mL^−1^) of Cr(vi) and NO_2_^−^ were prepared with K_2_CrO_4_ and NaNO_2_, respectively. Standard solutions of NO_2_^−^ (at pH 7.0) and Cr(vi) (at pH 4.0) were freshly diluted from respective stock solutions. Solutions of NaNO_3_, Na_2_CO_3_, NaHCO_3_, NaOH, NaHPO_3_, Na_2_PO_3_, Na_2_B_4_O_7_, PbCl_2_, CrCl_3_, CdCl_2_, CaCl_2_, MgCl_2_, MnSO_4_, NaCl and KCl were prepared at 100 μg mL^−1^, respectively. “Real-world” samples were collected from the Fen River (37.858055992N, 112.5312312824E), a local waste water pipeline (37.8595855992N, 112.5165812824E), a tap water pipe in the lab (37.861355992N, 112.5181812824E) and a home fish tank. Their pH values were adjusted to either 7.0 (for NO_2_^−^ detection) or pH 4.0 (for Cr(vi) testing) with NaOH (1 M) and HCl (1 M). The chromogenic reagent for nitrite (Griess reagent) contains 50 mM sulfanilamide, 330 mM citric acid and 10 mM *N*-(1-naphthyl)ethylenediamine in methanol. The chromogenic reagent for Cr(vi) was prepared with 0.2% 1,5-DPC and 1.0% methyltrioctylammonium chloride in acetone. Unless otherwise noted, all solutions were prepared with deionized water (>18.2 MΩ cm), produced from a Barnstead EasyPure Water System (Barnstead/Thermolyne, Dubuque, IA), both standard solutions and water samples were kept at 4 °C in the dark prior to the measurements.

### kPADs fabrication

**Fig. 1:**
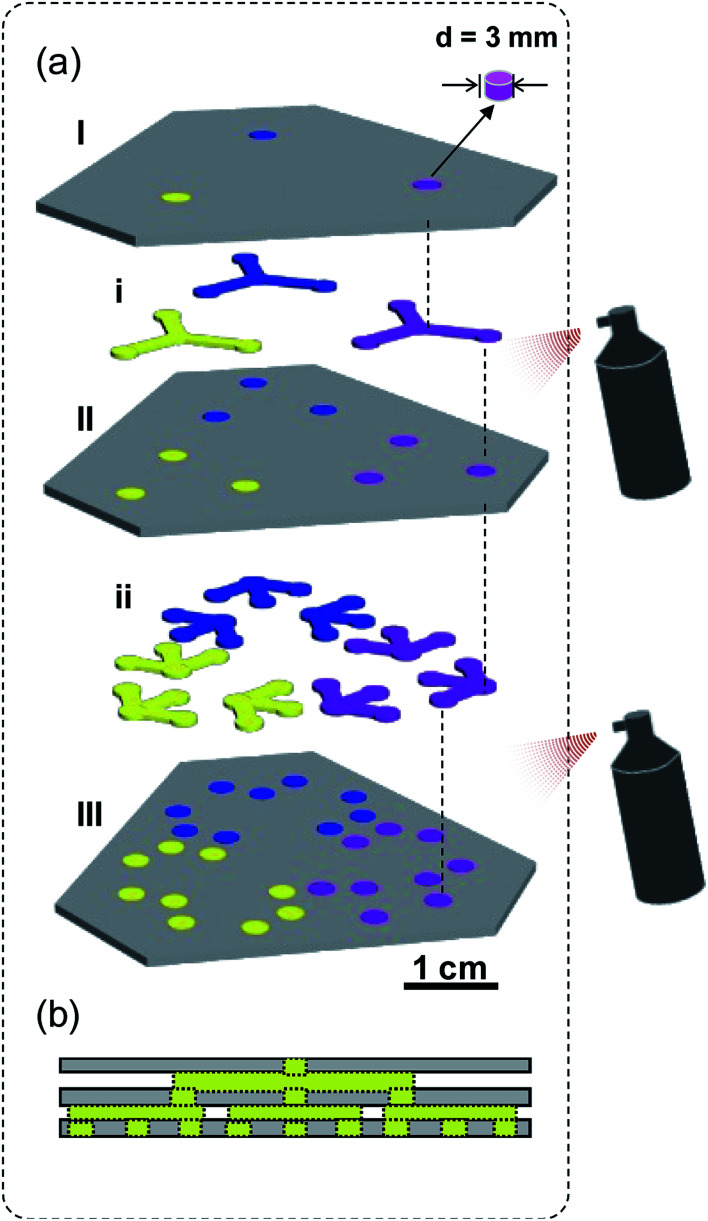
(a) Schematic view of the cut-and-paste fabrication of a kPAD. Color-coding: superhydrophobic hollow pads – dark gray; chromogenic reagent treated discs – blue, yellow, and pink; pre-cut hydrophilic discs and channels – light gray. (b) Cross section of the kPAD showing the “pathway” from one inlet to 9 outlets. The detailed fabrication procedure was presented as Fig. S1 in ESI.†

The fabrication procedure is fairly simple. At first, the patterns of hydrophilic channels and hollow pads were precisely designed (drawn) with a mapping software program (CorelDRAW X4) and printed using an office inkjet printer (EPSON R270) on filter paper (Whatman Grade 3). A pair of scissors and a hole puncher were used to cut the hydrophilic channels, hollow pads, and discs from the printed filter paper by carefully following the printed lines (to ensure the correct size and shape). Then the hollow pads (I, II, and III) were immersed in the binary solution of OTS and MTS (v/v, 3 : 7) in hexane at room temperature for 7 min and dried in an oven for 5 min at 40 °C, to achieve surface superhydrophobicity,^31^ followed by embedding its holes with pre-cut paper discs (*d* = 3 mm). Discs, embedded in the pad III, were cut from Grade 4, and others are cut from Grade 3 filter paper. Afterwards, hydrophilic channels (i, ii) were sandwiched between hydrophobic pads, which connect the hydrophilic discs in adjacent hydrophobic pads. As shown in Fig. 1a, all five layers (I, i, II, ii, and III) were vertically aligned and stack together by spraying a chemically stable adhesive (3M Super 77 Multi-Purpose) on II and III. To ensure the aqueous solution can flow smoothly, a minimal amount of spray adhesive should be carefully applied at the edges of each layer (see Fig. S1† for detailed fabrication procedure). The entire fabrication process can be accomplished within 10 min; the constructed kPADs are stored in a refrigerator (at 4 °C in the dark) prior to further experiments.

### Mobile app development

**Fig. 2:**
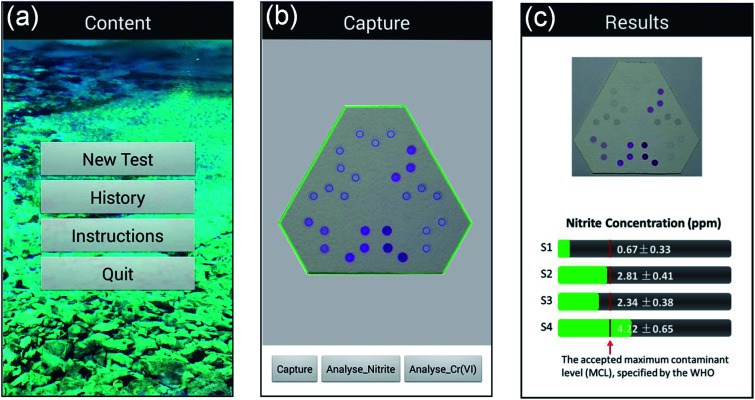
Screen snapshots of the “kPAD_DET” app running on an Android smartphone. (a) Launching menu; (b) measuring/testing menu; (c) reporting (result output) menu.

To read and analyze the colorimetric results developed on kPADs, a mobile app (named “kPAD_DET”), was developed with Android Developer Tools using Java, and installed on a Huawei G7 smartphone (the original code is provided as an ESI file†). As shown in Fig. 2, the main interface of the kPAD_DET has four options: New test, History, Instruction, and Quit (Fig. 2a). After “New test” is chosen, the rear camera of smartphone turns on and the preview window with a hexagonal green frame and twenty-seven blue circles appeared, which can be precisely aligned with the assay zones of kPADs. Then, the detection result was captured (Fig. 2b). The captured image and calculated concentrations of target analytes were displayed on the results interface (Fig. 2c). During the process of analysis, original green values of corresponding samples were obtained from the captured image of kPAD's detection zones (8 × 8 pixels, in the center of detection zone); then the least-squares method was used to fit the correlation between green value and corresponding concentrations. With the fitting equation, the concentrations of the unknown/spiked samples were calculated. To eliminate the influence of ambient lights, all measurements were performed in dark with a surface light source (145 Lux) aligned 45° obliquely above the kPAD, and the vertical distance was kept at 40 cm.

## Results and discussion

Two types of filter paper, Whatman Grade 3 and Grade 4, were used to prepare the kPADs. The kPAD shown in Fig. 1a consists of three layers of superhydrophobic pads (I, II and III, color-coded as dark gray) and two layers of pre-cut hydrophilic channels (i and ii, color-coded as light gray). Superhydrophobic layers (I and II) were reassemble with the precut “discs” (light gray, cut out form the same filter paper before the silanization treatment). While the pad III was “refilled” with paper discs cut from Whatman Grade 4 filter paper, which serve as the detection zones (color-coded in red, blue, and yellow for showing the complexity). Hydrophilic paper channels (i and ii), which allowed the solution to migrate through the layers (from I to III), were sandwiched between the superhydrophobic pads (I–III). As minimal amount of spray adhesive was used at the edge only, the aqueous solution can flow smoothly and reach the detection zones in the III layer. Without further miniaturization, the completed kPAD has a size similar to a business card. Detailed fabrication procedure of such a kPAD with three inlets and 27 outlets is presented in the ESI (Fig. S1).† Notably, the discs of detection zones were made of Whatman Grade 4, to obtain the optimum liquid flow between different layers (see Fig. S2† for optimization selection of the filter paper type). The hydrophobic pads were prepared with Whatman Grade 3, as it can be modified to be superhydrophobic when treated with a binary silane solution (OTS/MTS in toluene, 0.2% v/v).^31^ All other untreated paper channels and discs were prepared with Whatman Grade 4 filter paper to simplify the fabrication procedure.

Differing from the method reported by Martinez *et al.*,^14^ in which the “holes” were filled with cellulose powder, we utilized the original paper discs cut out from the same piece of filter paper; this was to ensure the best fit between the hydrophilic reaction zone and the hydrophobic “barrier”. With this design, the natural delivery of liquid samples across different layers remained and the second-step patterning is no longer needed. Compared with those devices created by directly folding or sticking two layers together,^14,15^ our design, inserting a “channel” layer between two pads ensures reliable transport of solutions within the 3D device. Very recently, Cardoso *et al.* have explored the use of readily available scholar glue to create hydrophobic flow barriers, and the glue-coated paper was then exposed to UV/Vis light for cross-linking to maximize chemical resistance and stability.^7^ Conceptually different from their innovation, herein we applied a minimal amount of adhesive spray, which is chemically stable and insoluble in water,^32^ to assemble different layers of μPADs. A detailed comparison between the kPADs with other existing 3D μPADs in terms of equipment requirements, patterning method, and assembly approach has been presented as Table S1 in the ESI.†

In comparison with the digital cutting-based xurography approach,^25^ which has been demonstrated to be powerful in creating 3D microfluidic devices with various polymeric materials (*e.g.*, micromixers and micromixing arrays),^26,27^ the cut-and-paste fabrication of kPADs promises a bench-top, instrumentation-free method to make disposable, paper-based devices for on-site or in-field applications (*vide infra*). It should be noted that the size, shape, and number of hydrophobic discs (reaction zones) can be flexibly altered to meet any practical demands, *i.e.*, the multiplicity and throughput can be adjusted as needed. The reproducibility of fabrication procedure and the stability of thus prepared kPADs have been confirmed (as shown in Fig. S3 and S4,† respectively). Particularly, the sizes of the holes on the hollow paper substrates and the cut-out discs show excellent correlations with the preset dimensions (*i.e.*, sizes of hole punchers), and can be prepared with high reproducibility (Fig. S3†).

**Fig. 3:**
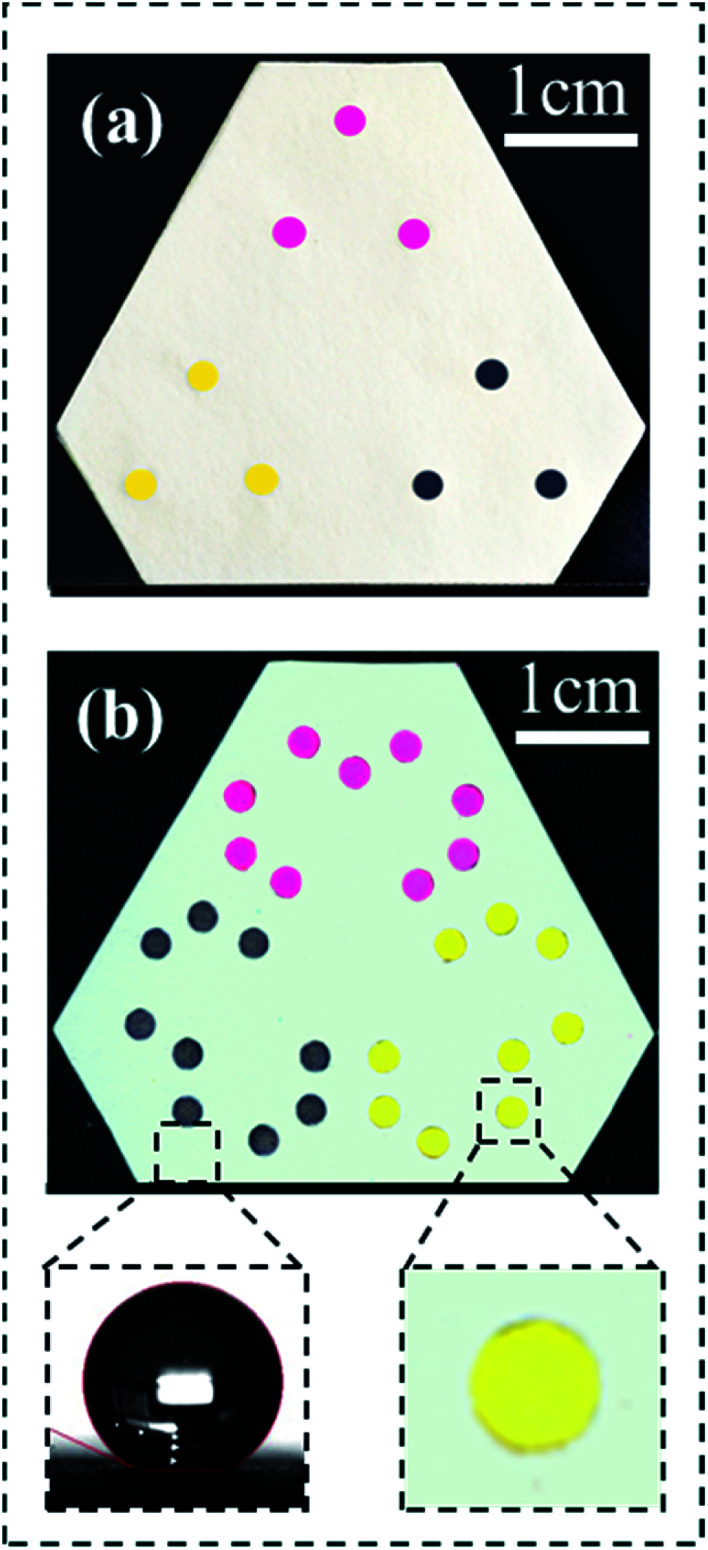
Validation experiment with diluted print ink solutions on the kPAD. (a) Photo showing the front side of the kPAD and (b) the back side. The bottom insets show a near-spherical water droplet (the water contact angle > 150°) on the hydrophobic pad (left) and an enlarged outlet (wicked with the yellow ink uniformly).

We have demonstrated the function of thus fabricated kPAD by adding 20 μL of 1 : 5 diluted inks (magenta, yellow, and black inkjet printer inks) to each of the inlets (Fig. 3a), and examining the color uniformity at corresponding outlets. As shown in Fig. 3b, all 27 outlets were wetted with respective ink in about 20 s, which is 20 to 50 times faster than previously created 3D μPAD (for which 5 to 15 min was needed for the solution to wet through, Fig. S5†).^14,22^ The facile transfer of solutions between different layers in our devices is due to the fact that we have retained the original porosity of filter paper. This is certainly an improved alternative from the compressed cellulose powder filling or UV patterning adapted in previous studies.^14,22^ The bottom right inset of Fig. 3b shows a zoom-in view of the outlet, which reveals the uniform color distribution across the entire reaction zone. The bottom left picture shows a water droplet on the modified paper base, whose contact angle was determined to be 155 ± 2° (which is superhydrophobic).^31^ In the case of wax or photoresist was used to pattern the paper base (to build hydrophobic barriers),^14–16^ typically water contact angles of slightly above 90° were achieved. Clearly, a device with patterned superhydrophobic substrate and unaltered paper disc shows optimized refinement in the reaction zone yet maintains the ideal liquid flow properties in the 3D configuration. The stability and solvent-resistance of these kPADs are guaranteed by the silanization treatment, which has been demonstrated to produce a structurally robust, superhydrophobic surface coating.^31^

The next step is to test the analytical performance of the kPADs fabricated *via* the cut-and-paste protocol for running quantitative assays that have potential applications. As the trial experiments, two environmental contaminants Cr(vi) and NO_2_^−^, representative heavy metal and inorganic salt, were chosen as they are typical sanitary contaminants in water. Long term intake of them both have persistence toxicity to human being, and likely results in esophageal cancer and gastric cancer.^33,34^ The detection of Cr(vi) was traditionally based on the colorimetric reaction between 1,5-diphenylcarbazide (DPC) and Cr(vi), which generates a magenta product [Cr(iii)DPCO]^(3−*n*)+^.^35–37^ While the NO_2_^−^ concentration in water samples was quantitated with the Griess reaction, which also generates a magenta product (the principle of colorimetric reaction were shown in Fig. S6†).^38–40^ One of the questions that should be addressed is how to correlate the concentration of analytes with the color information in the assay image, *i.e.*, which color should be analyzed for quantitation. The *M* (magenta) value from the CMYK (Cyan-Magenta-Yellow-Black) color space is naturally a good option to analyze the magenta products in both assays of Cr(vi) and NO_2_^−^. However, the picture acquired from a phone camera is based on the RGB (Red-Green-Blue) color space; besides, transferring an image format from RGB to CMYK may cause color loss.^41^ Herein, we directly use the original image (in RGB color space) for the analysis,^41–44^ which is based on the linear relationship between RGB to CMYK color spaces (eqn (1)).^42^ In practical applications, CMYK values usually use normalized expression (100%), *i.e.*, the value obtained from eqn (1) divided by 255.*C* = 255 − *R*

1 *M* = 255 − *G*

*Y* = 255 − *B**K* = min (*C*, *M*, *Y*)

The feasibility of using *G* (green) value to represent the magenta (*M*) intensity was verified by comparing the *G* and *M* values of the same color. In this case, different magenta dots were printed on a piece of filter paper (Whatman Grade 4), and then analyzed with the phone app. A linear relationship between *M* intensities and corresponding *G* values is present in the ESI (Fig. S7 and S8†), which is in good agreement with the fact that magenta and green are complementary colors.

**Fig. 4:**
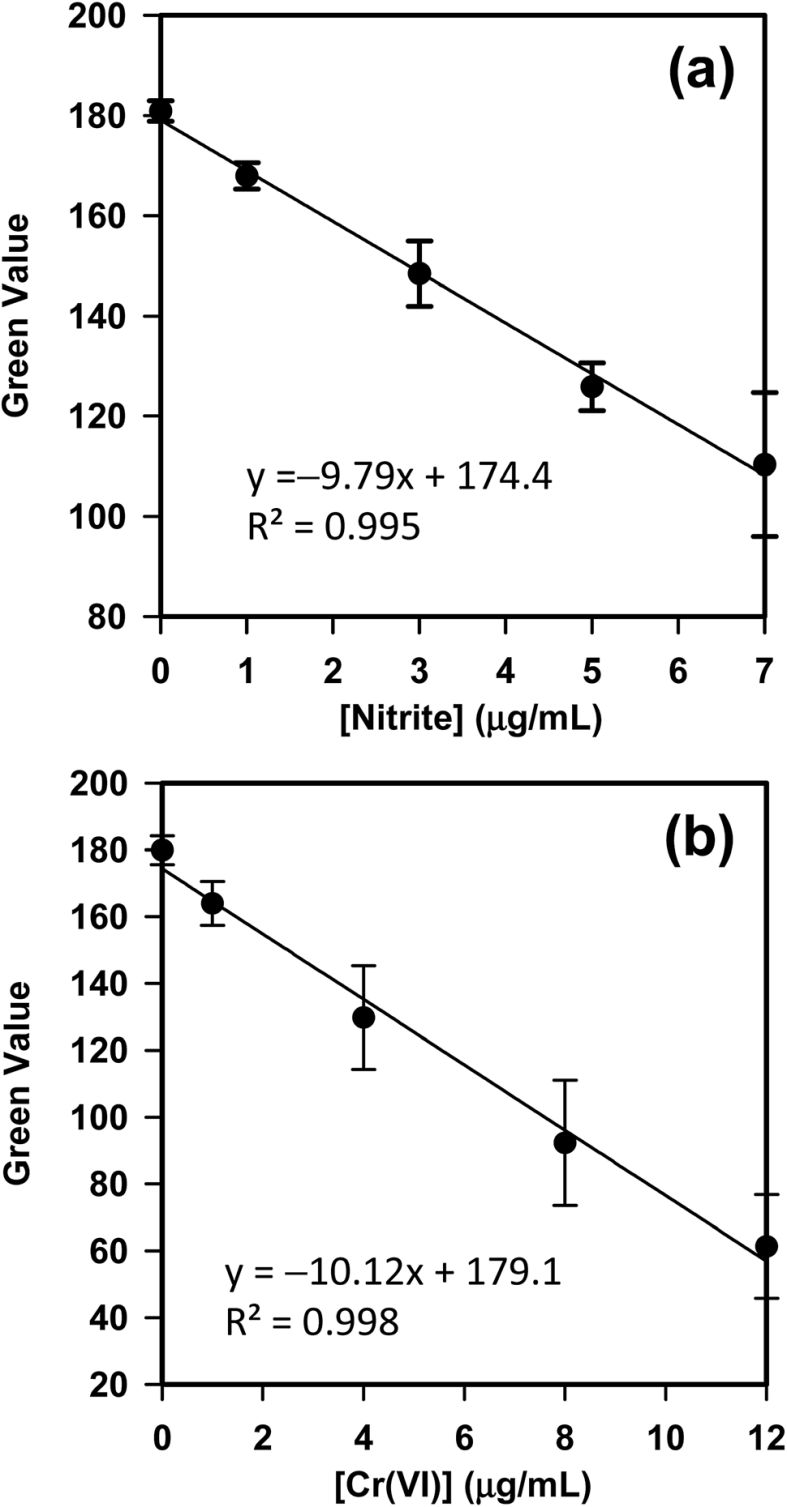
Calibration curves constructed with the mobile app (kPAD_DET) for the detection of (a) nitrite and (b) Cr(vi) on kPADs, respectively.

Due to the high-quality camera optics and open-source operation system, smartphones with mobile apps have been recently explored for signal reading and data analysis of colorimetric and scanometric assays.^45–47^ Hence, both assays of Cr(vi) and nitrite, conducted with kPAD, have been analyzed with our customized app. Nonetheless, comparative studies with standard spectrophotometric method have been carried out to optimize the reaction conditions and to validate the reliability of kPADs methods (see Fig. S9 in the ESI†). For the detection of Cr(vi) and nitrite carried out with kPADs, five standard solutions were used in each case to construct the calibration curve and four target samples were tested, respectively. The detection zones (outlets) on a kPAD were pretreated with the chromogenic reagent for either nitrite or Cr(vi) prior to the final assembly step (selectivity tests for nitrite and Cr(vi) were shown in Fig. S10†). As shown in Fig. 4, the calibration curves established based on the correlation between the *G* value and the concentration for both NO_2_^−^ and Cr(vi) standard solutions showed perfect linear relations with *R*^2^ values of 0.995 and 0.998. The detection limits for Cr(vi) and NO_2_^−^ were calculated to be 0.7 μg mL^−1^ and 0.4 μg mL^−1^, respectively, according to the “3*σ*/*b*” rule (where *σ* is the standard deviation of the *y*-intercept of the regression line and *b* is the slope). These values are comparable with the detection limits obtained with other colorimetric assays reported in literature.^34,37^ Nevertheless, these calibration curves and the deduced equations for the signal–concentration correlations were incorporated to the kPAD_DET app that can be directly applied for the quantitation of unknown samples (*vide infra*).

**Fig. 5:**
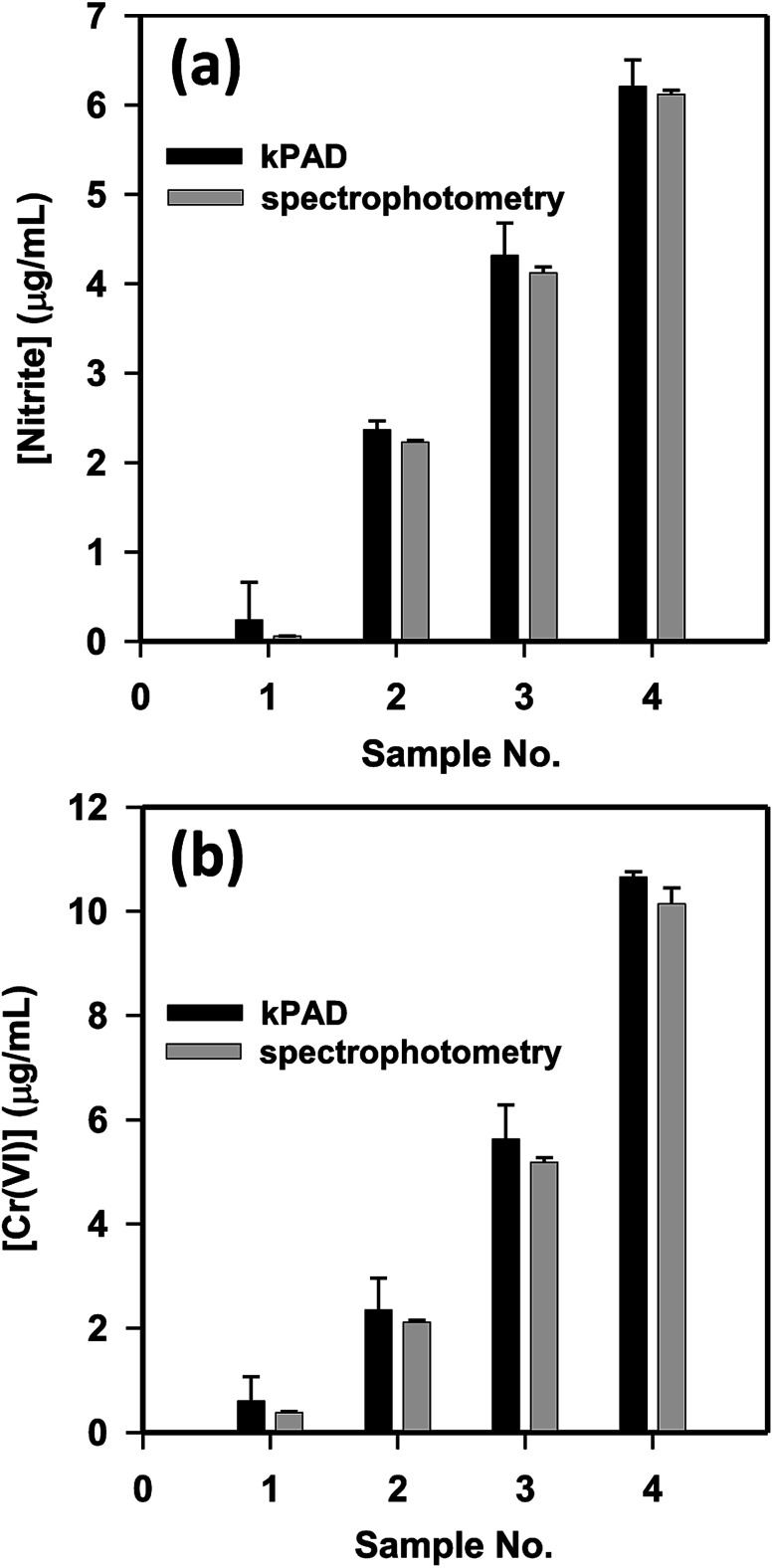
Comparison of the kPAD and conventional spectrophotometry methods for the detection of nitrite and Cr(vi) in tap water. (a) Samples 1–4 are tap water spiked with 0, 2, 4, and 6 μg mL^−1^ of sodium nitrite, respectively; (b) samples 1–4 are tap water spiked with 0, 2, 5, and 10 μg mL^−1^ of potassium dichromate, respectively.

Upon sample testing, we capture a picture of the outlet-side of the kPAD (Fig. 2b) and analyze the results with the mobile app. For proof-of-concept, the concentration of nitrite and Cr(vi) of tap water was detected by spiking standard nitrite (0, 2, 4, 6 μg mL^−1^) and Cr(vi) (0, 2, 5, 10 μg mL^−1^) solutions, respectively; meanwhile comparative detections have been carried out with a standard spectrophotometer. For the detection of NO_2_^−^ and Cr(vi) with the spectrophotometry method, the amount of chromogenic reagent used were respectively 400 μL and 60 μL for every 2.0 mL standard or sample solution. In contrast, as little as 20 μL-standard or sample solutions were needed for the kPAD detection. As shown in Fig. 5, the concentrations determined with the “kPAD_DET” system correlated well with those obtained with a spectrophotometer within the experimental uncertainties, and were both close to the actual concentration (spiked value). The statistical consistency between the two methods (Fig. S11†) further confirms the reliability of the kPADs detection over a wide range of analyte concentrations.

**Fig. 6:**
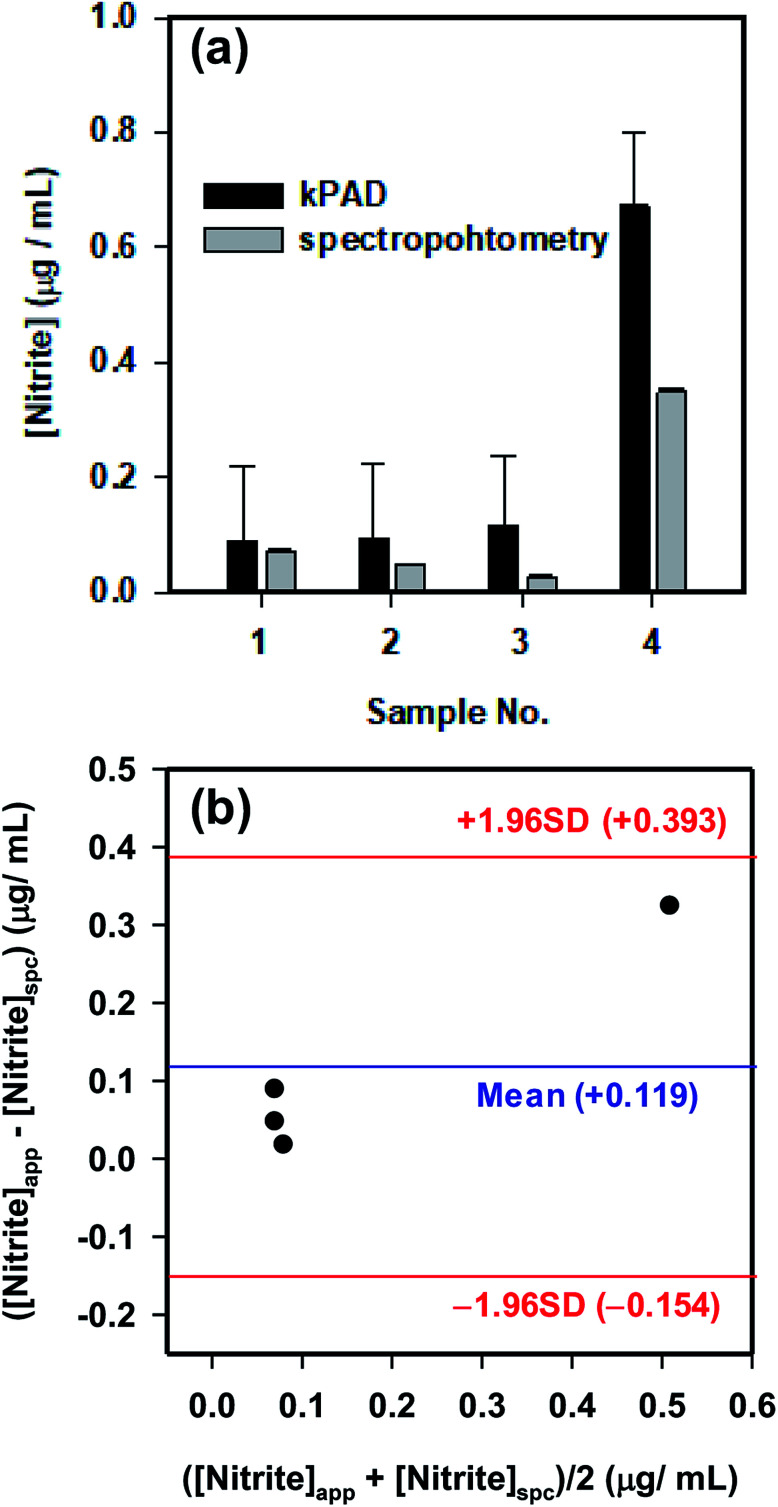
(a) The detection of nitrite from real world water samples on a kPAD, and its comparison with the conventional spectrophotometric results. Samples 1–4 represent river water (Fen River, Taiyuan, Shanxi), fish tank water, waste water, and tap water, respectively. Black bars are the data obtained from standard spectrophotometric measurements, and the grey bars are the results from the kPAD. (b) Bland–Altman plot showing the agreement between the determined nitrite concentrations with the kPAD and standard spectrophotometric methods.

To further validate the application potential of the kPADs fabricated with the simple cut-and-paste method, the concentration of NO_2_^−^ in several “real-world” samples were tested and compared with the standard spectrophotometry method (Fig. 6a). The concentrations determined with the kPAD_DET system are again in good agreement with those obtained with spectrophotometry measurements. As a “gold standard” method,^48^ we have further shown the satisfactory comparison between the measurement techniques with the Bland–Altman plot (Fig. 6b). While the detection limit determined above (0.4 μg mL^−1^, Fig. 4a) meets the primary drinking water standard (1.0 μg mL^−1^) stated by the US Environmental Protection Agency (EPA),^49^ we in fact can quantitate much lower concentration of NO_2_^−^ (from 0.03 to 0.4 μg mL^−1^, as shown in Fig. 6a); this is in fact much lower or at the same order of magnitude of the nitrite concentration in typical drinking water (0.1 μg mL^−1^) as reported by the World Health Organization (WHO).^50^ It is not debatable that the traditional spectrophotometric method provides greater sensitivity and accuracy (with rather smaller experimental uncertainties as shown in Fig. 5a and 6a), which requires advanced instrumentation and professionally trained operators. These pre-assembled kPADs are portable and disposable; they are suited for applications in field tests or other remote setting, as the only requirements would be samples of interests and a smartphone with the installed app. That is to say, the low cost, sufficient sensitivity and ease of use highlight the potential of the cut-and-paste protocol to prepare kPADs for rapid, on-site colorimetric assay applications, where traditional analytical instrumentation is not readily accessible. While we have shown the capability of kPADs for the quantitation of two representative environmental contaminants in water samples, the application can be readily expanded to a broad array of analytes, by simply transforming established colorimetric reactions to paper substrates.

## Conclusion

It has been demonstrated that the ancient papercutting art, *kirigami*, promises a novel cut-and-paste method for benchtop fabrication of 3D μPADs with excellent liquid handling capability, and that these *kirigami* μPADs (kPADs) can be integrated with a mobile app for performing quantitative colorimetric assays. The preparation of the kPADs is fast, inexpensive, and does not require any special photolithographic devices or masks, for which they are suitable for implementing quantitative analysis for remote sites or resource-limited regions. In conjunction with a custom mobile app, we have demonstrated the application of these kPADs in quantitative, colorimetric analysis of water samples, *i.e.*, colorimetric assays for both nitrite and Cr(vi) have shown excellent agreement with the results of standard spectrophotometric measurements.

## Conflicts of interest

A China Patent (ZL 201510377398.5) was granted on May 3, 2017 for which the inventors are Xiaochun Li, Jianhua Wang, and Hua-Zhong Yu. The patent covers the fabrication and application of kPADs described herein. The inventors have begun dialogues with a number of biotech companies to explore the commercialization of such devices.

## Supplementary Material

RA-009-C9RA04014E-s001

RA-009-C9RA04014E-s002
