# Supplementary material for: From kirigami to three-dimensional paper-based micro-analytical device: cut-and-paste fabrication and mobile app quantitation
Source: RSC Adv. 2019 Jul 26;9(40):23267–75. doi: 10.1039/c9ra04014e (PMC9067308; doi:10.1039/c9ra04014e)
Supplement: RA-009-C9RA04014E-s002 [file RA-009-C9RA04014E-s002.pdf]

Electronic Supplementary Material (ESI) for RSC Advances.
This journal is © The Royal Society of Chemistry 2019

Electronic Supplementary Information (ESI)

for

From Kirigami to Three-dimensional Paper-based Micro-analytical Device: Cut-and-paste Fabrication and Mobile App Quantitation†

Jianhua Wang,[a‡] Lishen Zhang,[b‡] Xiaochun Li,[a*] Xiaoliang Zhang,[a] and Hua-Zhong Yu[ab*]

[a] College of Biomedical Engineering, Taiyuan University of Technology, Taiyuan, Shanxi 030024, China

[b] Department of Chemistry, Simon Fraser University, Burnaby, British Columbia V5A 1S6, Canada

[*] E-mail addresses: lixiaochun@tyut.edu.cn (X.L.); hogan_yu@sfu.ca (H.Y.)

[‡] These authors contributed equally to this work.

Detailed experimental procedure for the cut-and-paste fabrication of kPADs; optimization of the filter paper type; fabrication reproductivity and device stability tests; color intensity reading protocol; additional experimental details of the colorimetric detection of nitrite and Cr(VI), selectivity tests, and statistic comparison with conventional spectrophotometric methods and literature results.

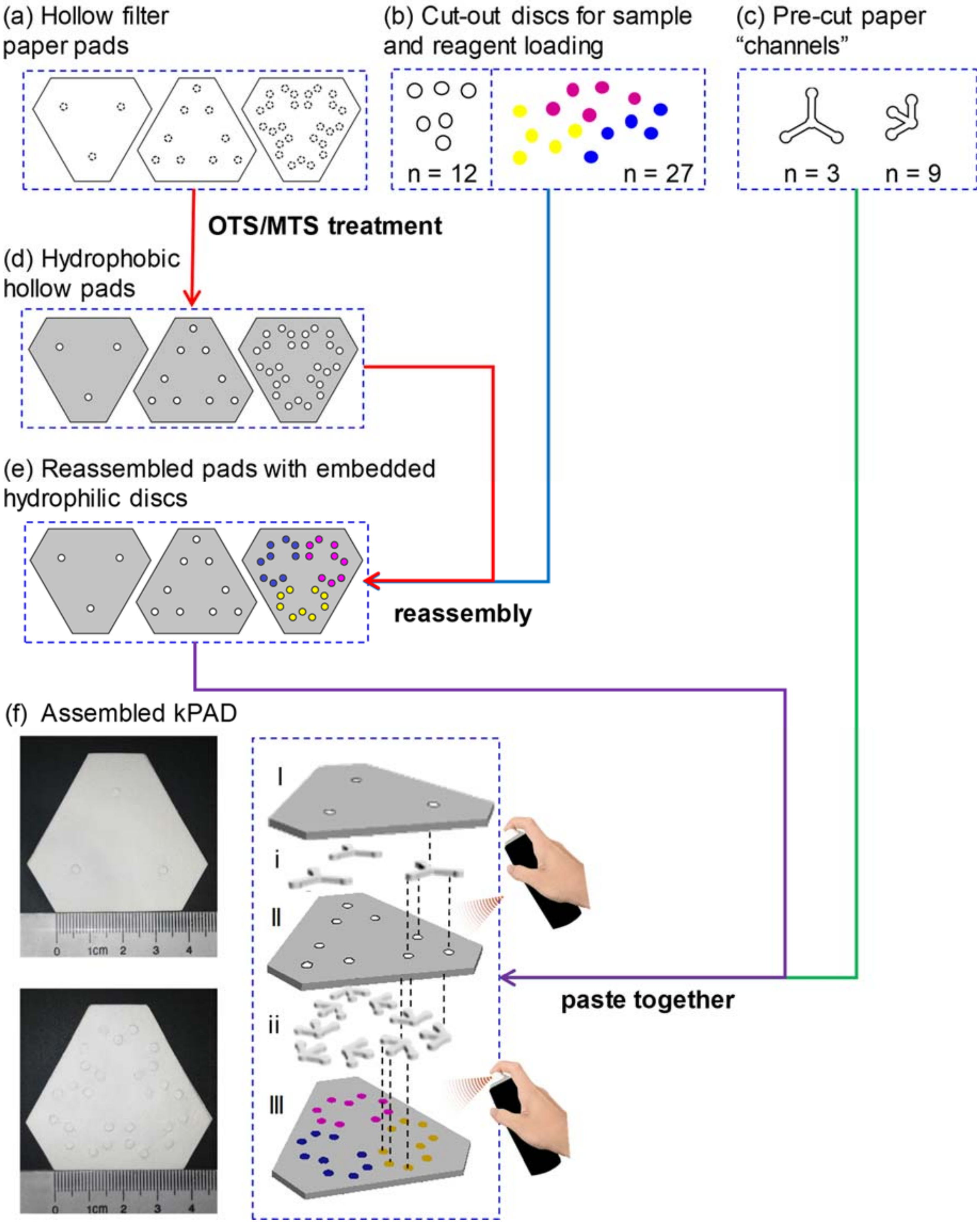

Fig. S1. "Cut-and-paste" fabrication of kPADs from off-the-shelf laboratory filter paper upon silanization. The device is consisted of hollow filter pads (a), cut-out hydrophilic discs (b), and precut paper channels (c). The patterns of these three different parts were designed with a mapping software program (CorelDRAW X4) and printed using an office inkjet printer (EPSON R270) on regular laboratory filter paper (Whatman Grade 3). The three hollow pads were prepared from large pieces of filter paper; each was punched with a hole puncher to cut out 3, 9, and 27 paper discs (diameter = 3

mm), respectively. The untreated paper discs will be used for both sample delivery (color coded in white) and chromogenic reagents (color coded in yellow, red, and blue). A pair of scissors was used to prepare the hydrophilic paper channels from another large piece of filter paper; both the cutting and punching processes were finished along the printed lines to ensure the exact size and shape of the designed patterns, so as to avoid the misalignment of hydrophilic channels and detection zones in the assembled kPAD. The hollow pads were immersed in a binary solution of OTS and MTS (v/v, 3:7) in hexane room temperature for 7 min and dried in an oven for 5 min at 40 °C, to achieve the surface superhydrophobicity (d). Afterwards, the hydrophobic hollow pads were reassembled with the paper discs (e), and finally the hydrophilic channels were sandwiched between the hydrophobic pads, which connect the hydrophilic discs in adjacent hydrophobic pads. The pads, discs, and channels were vertically aligned and glued together by using a spray adhesive (3M Super 77 Multi-Purpose) (f).

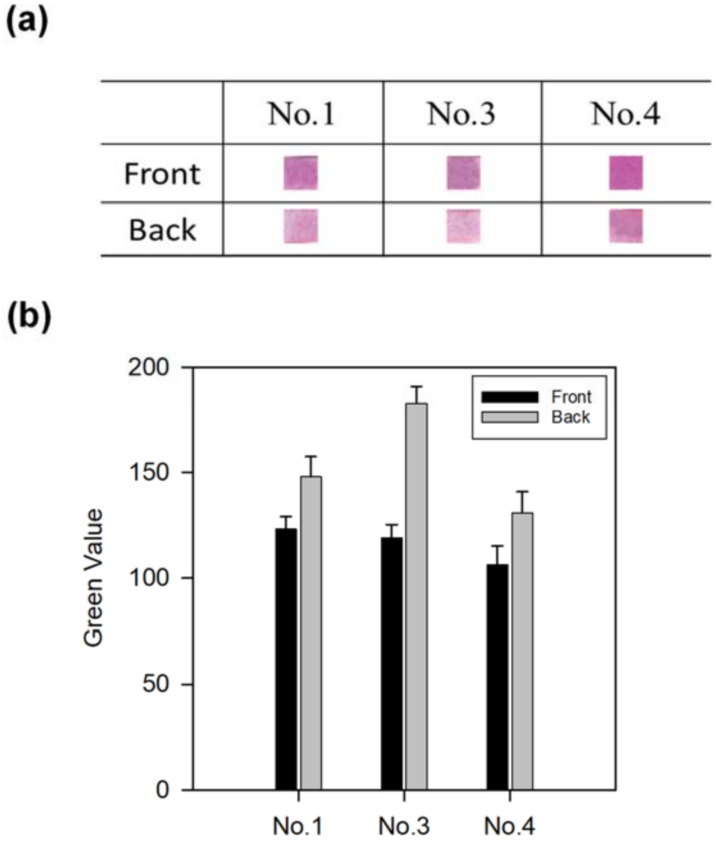

Fig. S2. Optimization of the filter paper type used for preparing kPAD. (a) Chromogenic results on front and back side of paper strips for the detection of Cr (VI) (10 μg/mL) on three common types of filter paper; (b) Comparison of the green values read from the assays prepared on three different types of filter paper shown in (a). Grade 1 filter paper (pore size: 11 μm; thickness: 180 μm) is typically used for traditional qualitative analytical separations. Grade 3 filter paper has a smaller pore size (6 μm) and doubles the thickness of Grade 1 (390 μm); it is typically used for the retention of finer particles and allows for more precipitate to be held without clogging. More importantly, this type of filter paper can be modified with OTS/MTS to be superhydrophobic (i.e., with a water contact angle of > 150°).[27] In comparison, Grade 4 paper has a large pore size (20-25 μm) and a thickness of 205 μm, with extremely fast liquid flow rate. Therefore, we have adapted Grade 3 filter paper for making the hollow paper pads (modified to be superhydrophobic) and Grade 4 for the discs (unmodified).

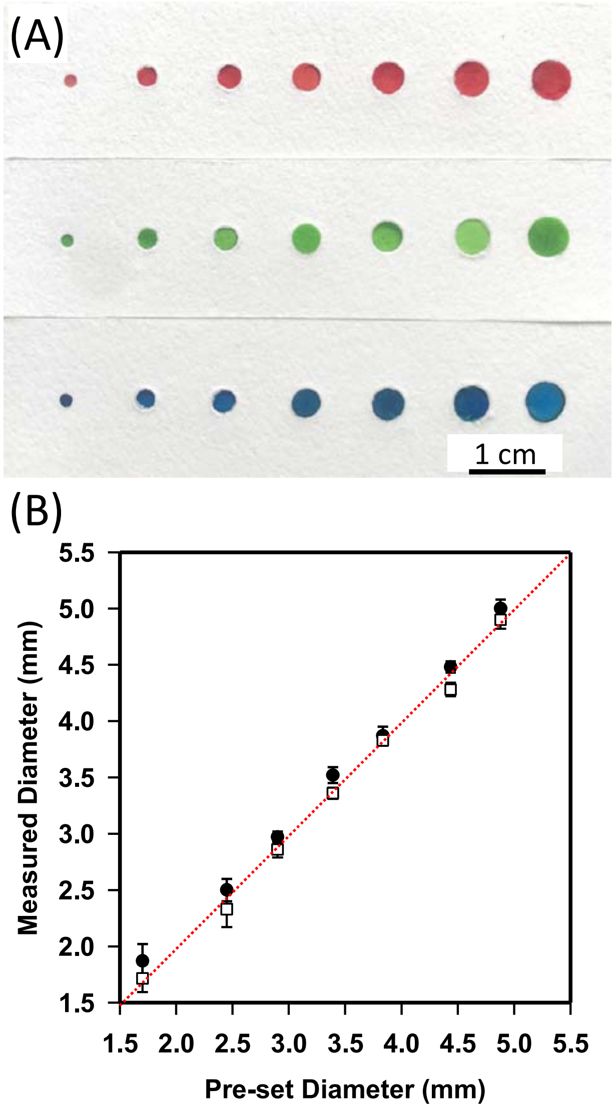

Fig. S3. Reproductivity of the fabrication of kPADs. (a) Three sets of punched discs assembled with hollow paper pads, the discs were dyed with different color for clear viewing. (b) The correlation between the size of the punched holes on paper pads (open squares) and that of the discs (filled dots). The key step to make a well performed kPAD is the hole punching and disc assembly process (Fig. S1); as shown in (b) we can reproducibly make paper discs with desired size (down to 1.5 mm in diameter), and they can be assembled to the hollow paper pad (treated to be superhydrophobic). The perfect matching between the hole and disc as well as the even distribution of colors on the discs upon assembly confirm that we can reproducibly make the kPADs with almost no variations as originally designed.

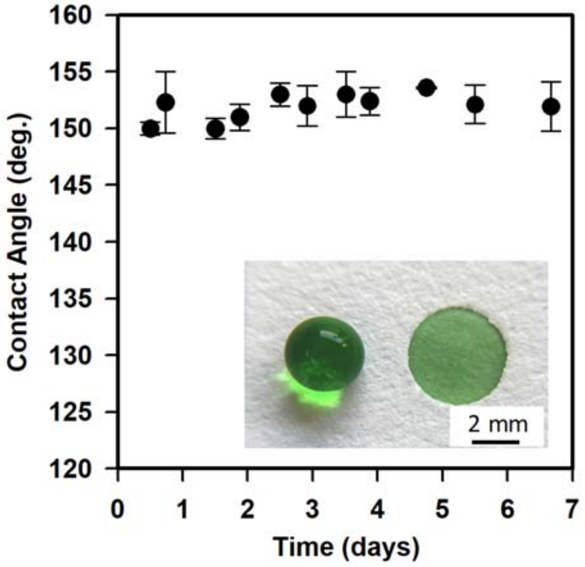

Fig. S4. Stability of fabricated kPADs upon prolonged storage. The inset shows a picture of part of the kPAD after one-week storage in dark, for which the embedded paper disc remains hydrophilic (wicked evenly by the green ink) and the hollow pad is still superhydrophobic. In fact, we have tested the device after 7 weeks and two years, and did not observe any discernible changes.

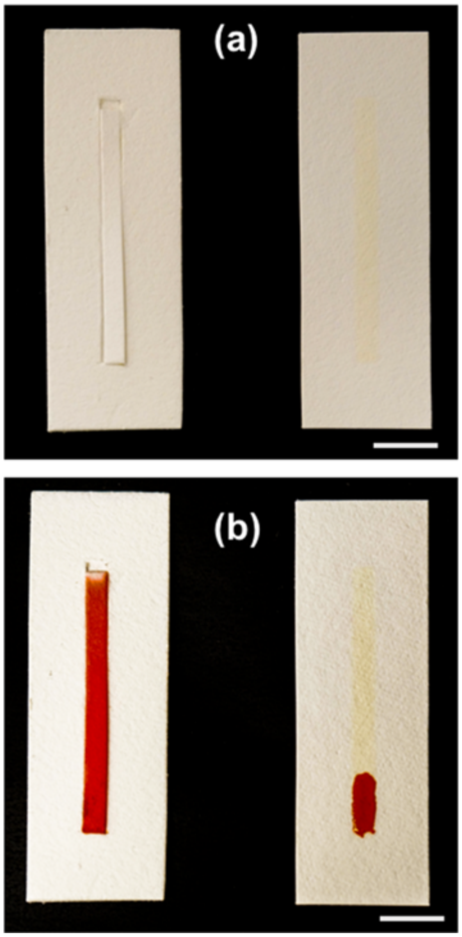

Fig. S5. Comparison of liquid flow rates on cut-and-paste prepared paper device (kPAD) and that prepared via UV activation. Water flow rate and background color comparisons of kPAD (left) and UV/ozone lithography treated PAD (right), before (a) and 20 s after (b) pipetting 30 µL of food dye solution, respectively. For both channels, the length is 4 cm and width is 0.3 cm. The scale bar is 1 cm. The time spent for food dye solution to reach the end of the kPAD channel is 20 s. However, for the UV/ozone lithography treated PAD, the solution did not reach the end before it totally evaporated (15 min). Also, kPAD channel keeps the original white color of the filter paper, but UV/ozone lithography treated PAD shows yellow background.

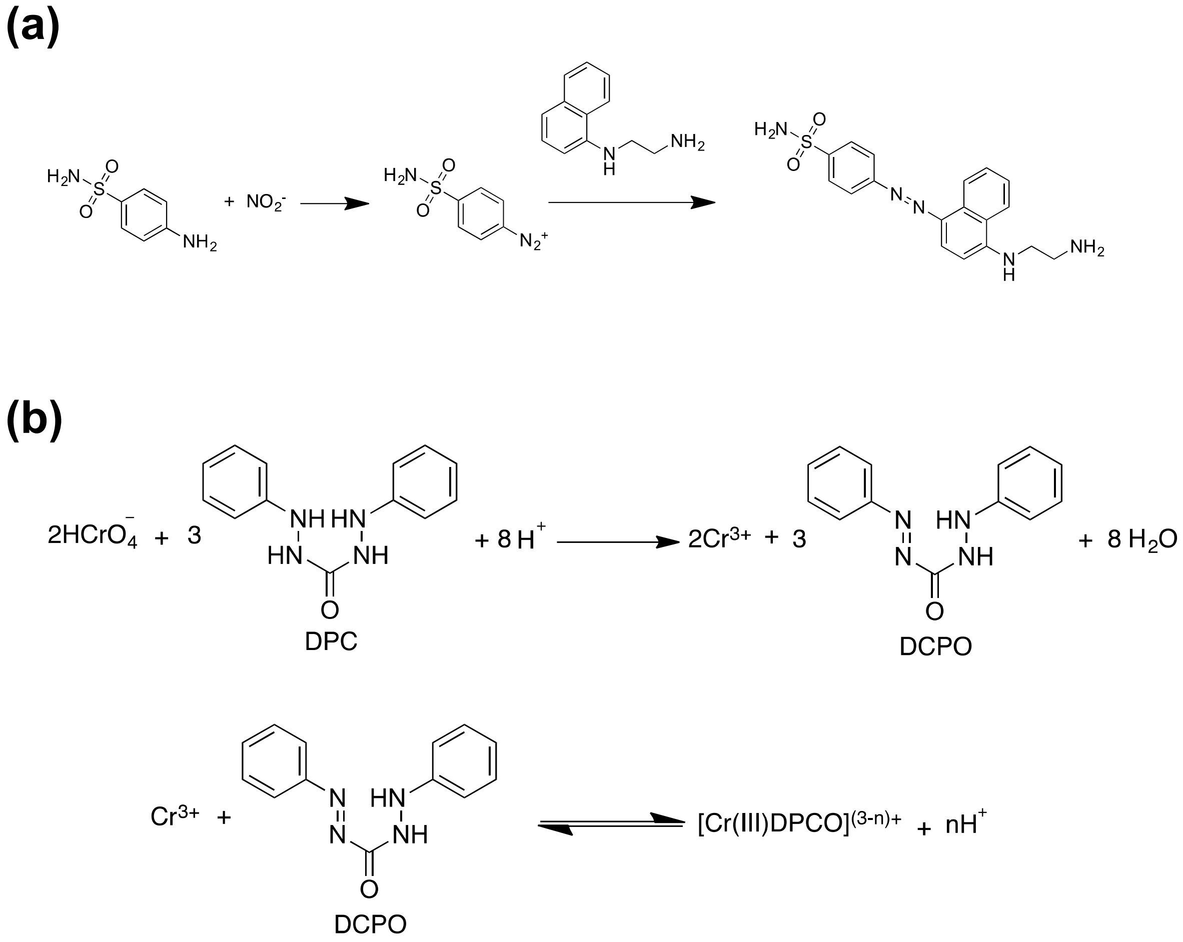

Fig. S6. (a) The Griess reaction for the detection of nitrite ions; (b) The colorimetric reaction for the detection of Cr(VI).

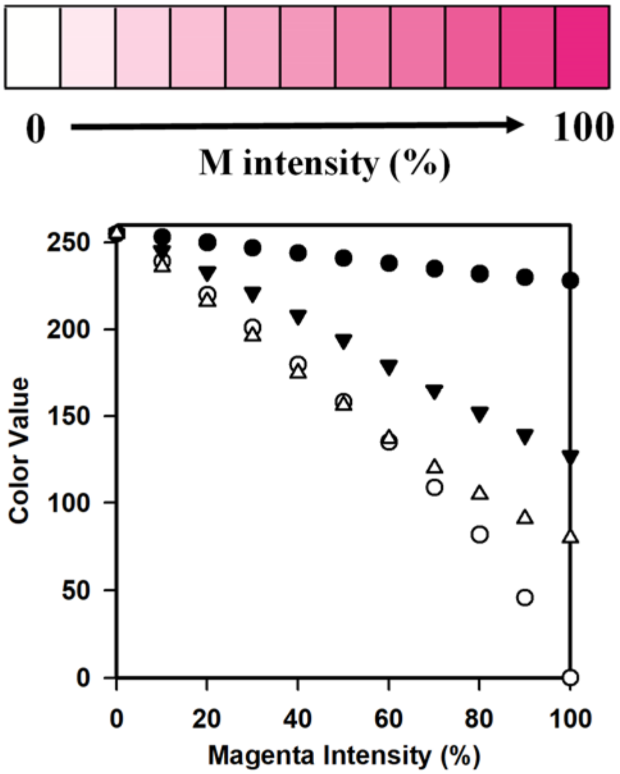

Fig. S7. Color strips designed by using Adobe Photoshop (CS2) with the M intensity ranging from 0% to 100% (values of C, Y, and K are all set to 0%); the relationship of R (●), G (△), B (▼), and grayscale (O) values with the M intensity of each of the color strip.

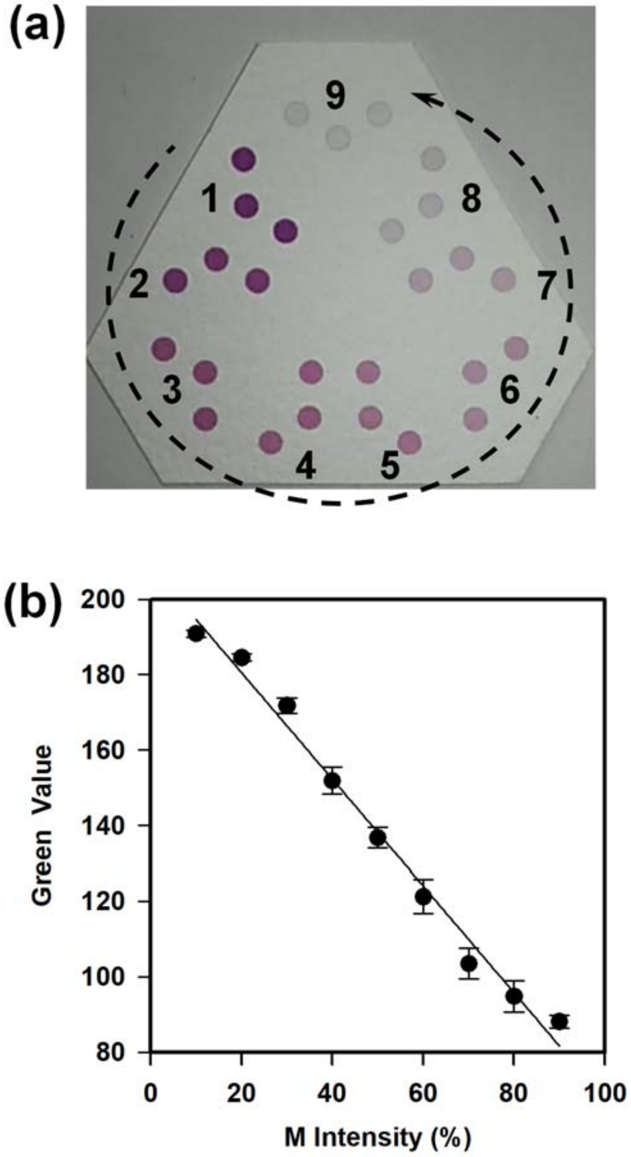

Fig. S8. (a) Photo of nine groups of dots (with different M intensity) printed on filter paper with the same size, shape, and relative position as the outlets of the kPAD; (b) linear correlation between the G values obtained with kPAD DET and the preset M intensity.

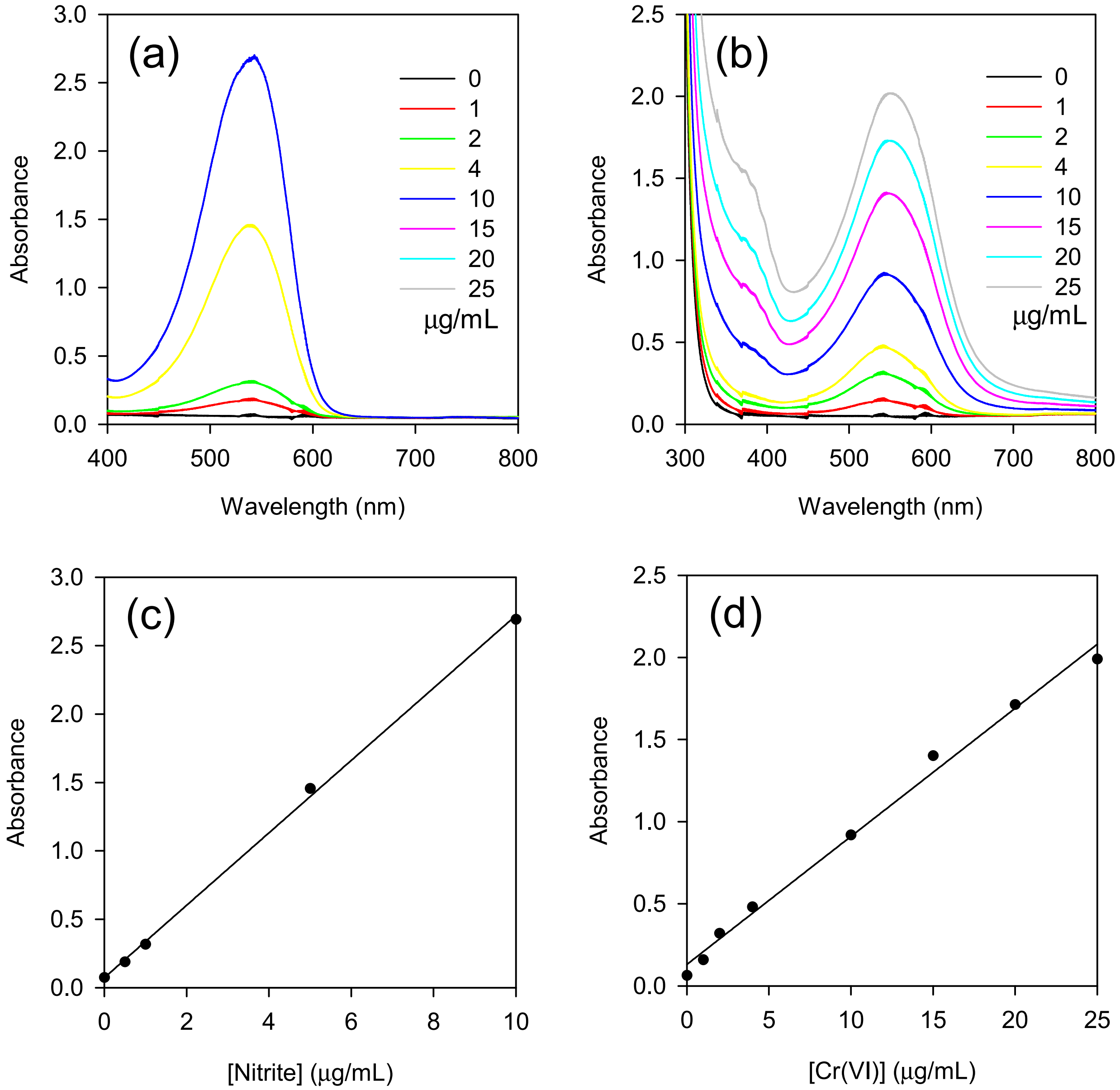

Fig. S9. UV-Vis spectra of the colorimetric reaction products with different concentrations of nitrite (a) and Cr(VI) (b); and thus, obtained calibration curves for nitrite (c) and Cr(VI) (d), respectively.

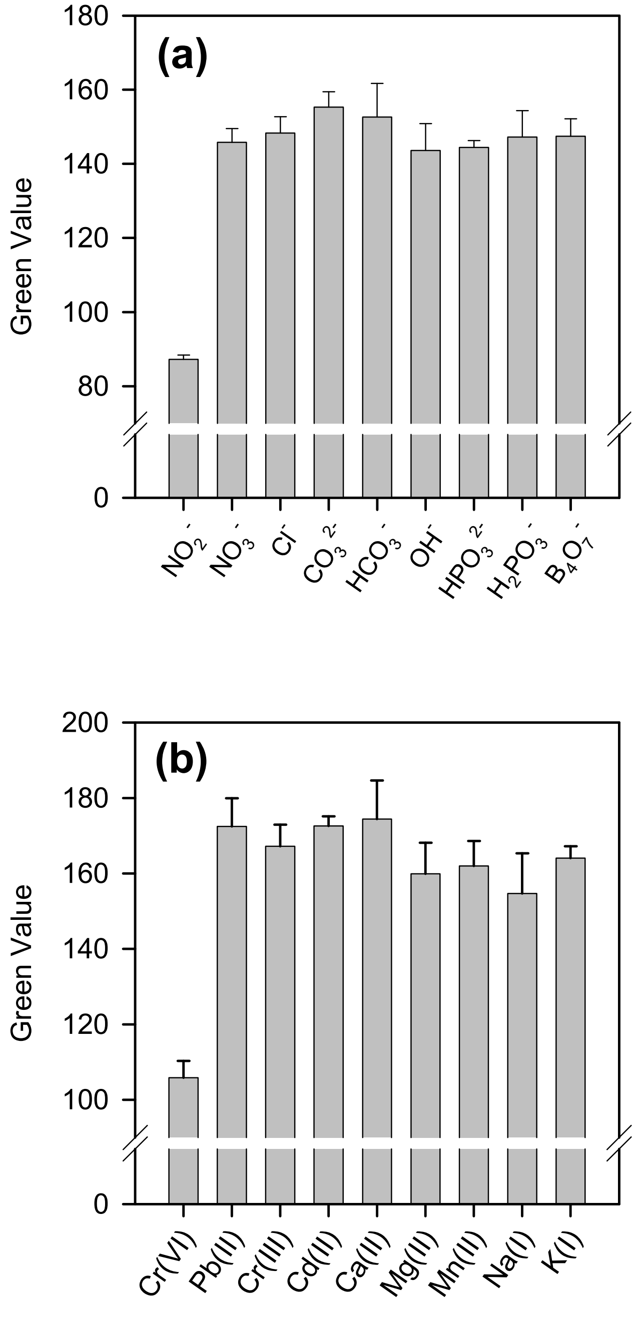

Fig. S10. Selectivity tests for (a) nitrite and (b) Cr(VI) with the kPAD (smartphone app reading). The concentration of the analyte (nitrite / Cr(VI)) was 10 μg/mL, while all other interfering ions were prepared at 100 μg/mL.

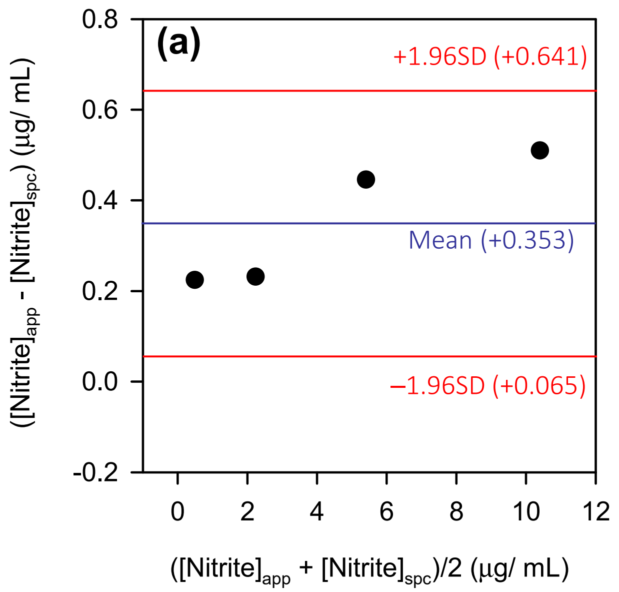

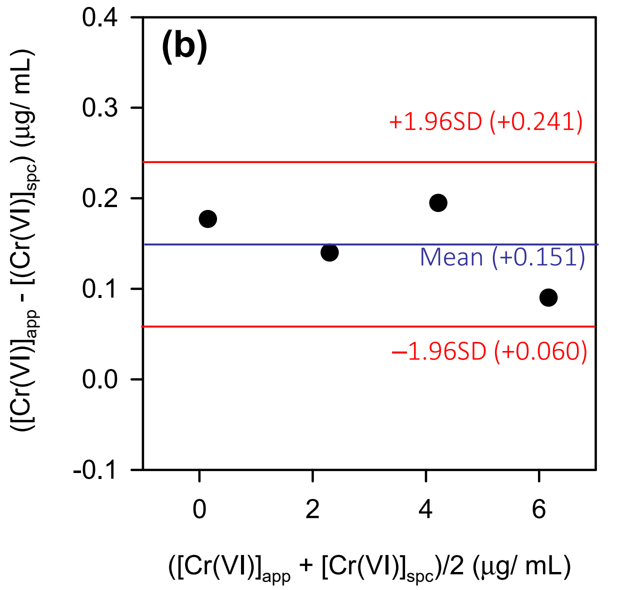

Fig. S11. Bland-Altman plots showing the agreements between the detection of (a) Nitrite and (b) Cr(VI) with the kPAD and standard spectrophotometric method. The data are based on the results presented in Figure 5 in the main text.

Table S1. Comparison of the kPAD fabrication with literature methods for preparing 3D µPADs.

Ref.	Fabrication equipment	Patterning method	Assembly method
14	UV lamp, hotplate, laser cutter	Cellulose powder filling	Stacking
15	UV lamp, hotplate	UV patterning	*Origami* (folding)
16	Wax printer, hotplate	Wax patterned channel	Stacking
22	Wax printer, oven	Cellulose powder filling	Stacking
23	Wax printer, oven, knife or scissors	Wax patterning	*Origami* (folding)
24	UV lamp; photolithograph masks	UV lithography of surface-coated photoresist	N/A
This work	Scissors, hole puncher	Hole punching, cutting (*Kirigami*)	Re-assembly / stacking

Table S2. Comparison of the limit of detection (LOD) for nitrite obtained with kPAD and those reported in literature.

Ref.	Limit of detection (LOD)
31	4.6 µg/mL (0.1 mM)
34	0.52 µg/mL
39	0.46 µg/mL (10 µM)
40	0.26 µg/mL (5.6 µM)
This work	0.50 µg/mL

Table S3. Comparison of the limit of detection (LOD) for Cr (VI) obtained with kPAD and those reported in literature.

Ref.	Limit of detection (LOD)
35	0.5 µg/mL[a]
36	0.12 µg (solid sample)
37	0.02–1.50 µg/mL[b]
This work	0.7 µg/mL

[a]: lowest concentration being tested and detectable; no calibration curve was constructed
[b]: detection range claimed, LOD not determined
